# Photodynamic therapy inhibits p-glycoprotein mediated multidrug resistance via JNK activation in human hepatocellular carcinoma using the photosensitizer pheophorbide a

**DOI:** 10.1186/1476-4598-8-56

**Published:** 2009-07-31

**Authors:** Patrick Ming-Kuen Tang, Dong-Mei Zhang, Ngoc-Ha Bui Xuan, Stephen Kwok-Wing Tsui, Mary Miu-Yee Waye, Siu-Kai Kong, Wing-Ping Fong, Kwok-Pui Fung

**Affiliations:** 1 School of Biomedical Sciences, The Chinese University of Hong Kong, Shatin, N.T., Hong Kong, PR China; 2 Department of Biochemistry, The Chinese University of Hong Kong, Shatin, N.T., Hong Kong, PR China; 3 Institute of Chinese Medicine, The Chinese University of Hong Kong, Shatin, N.T., Hong Kong, PR China

## Abstract

**Background:**

Multidrug resistance (MDR) is frequently observed after prolonged treatment in human hepatoma with conventional anti-tumor drugs, and photodynamic therapy (PDT) is a recently suggested alternative to overcome MDR. The therapeutic potential of PDT was evaluated in a multidrug resistance (MDR) human hepatoma cell line R-HepG2 with photosensitizer pheophorbide a (Pa).

**Results:**

Our results demonstrated that intracellular accumulation of Pa was not reduced by the overexpression of P-glycoprotein. Pa-based PDT (Pa-PDT) significantly inhibited the growth of R-HepG2 cells with an IC50 value of 0.6 μM. Mechanistic study demonstrated that genomic DNA fragmentation and phosphatidylserine externalization occurred where increase of intracellular singlet oxygen level triggers the phosphorylation of c-Jun N-terminal Kinase (JNK) and leads to activation of intrinsic apoptotic caspases cascade during the Pa-PDT treatment. The cytotoxicity of Pa-PDT, accumulation of sub-G1 population, and depolarization of mitochondrial membrane could be inhibited by JNK inhibitor in the Pa-PDT treated cells. Interestingly, the Pa-PDT induced JNK activation showed inhibitory effect on MDR by the down-regulation of P-glycoprotein in R-HepG2 cells in a dose-dependent manner. In addition, significant reduction of tumor size was obtained in Pa-PDT treated R-HepG2-bearing nude mice with no significant damages in liver and heart.

**Conclusion:**

In summary, our findings provided the first evidence that PDT could inhibit the MDR activity by down-regulating the expression of P-glycoprotein via JNK activation using pheophorbide a as the photosensitizer, and our work proved that Pa-PDT inhibited the growth of MDR hepatoma cells by mitochondrial-mediated apoptosis induction.

## Introduction

Photodynamic therapy (PDT) was applied to treat diseases such as psoriasis, rickets, vitiligo and skin cancer thousand of years ago by ancient Egyptian, Indian and Chinese [1-3]. PDT requires the presence of two non-toxic elements: photosensitizer and light irradiation. When they are applied together, a rapid intracellular stress is generated in target tissues. Photosensitizers usually produce reactive oxygen species (ROS) after receiving light energy during illumination in an oxygen-rich environment, and eventually initiate apoptosis or necrosis in the treated cells [4]. Recently, PDT has been proposed as an alternative to overcome multidrug resistance (MDR) for anti-cancer treatment [5].

MDR is a situation that cancer cells are able to evade the cytotoxic effects of a broad range of anti-tumor agents. Expression of ATP-dependent membrane transporter P-glycoprotein to pump drugs out of the cells is a common phenomenon in tumor cells with MDR phenotypes [6]. MDR frequently appeared in liver cancer patients after prolonged systemic treatment with anti-tumor drugs. For example, low response rates (15 to 20%) were found in hepatoma cases using doxorubicin (Dox) as an anti-cancer drug [7]. Furthermore, as P-glycoprotein in MDR tumor cells can pump out a broad range of structurally and functionally unrelated anti-cancer agents, it is difficult to treat MDR cancer patients by chemotherapy [8].

Hepatoma is one of the most common malignancies, which contributes approximately 5 to 10% of all cancer cases worldwide and nearly 1 million deaths annually. However, no adjuvant or palliative treatment modalities have been conclusively shown to prolong the survival of patients suffering from hepatocellular carcinoma [7]. Liver cirrhosis is a common cause of hepatoma and it is difficult for the surgical resection to treat hepatoma developing from this cause, accounting for less than 18% of this type of patients. Thus, local ablation, intra-arterial and systemic treatments are major therapeutic modalities for hepatoma [9]. Therefore, development of new agents with mild side effects and capability to circumvent the MDR in hepatoma cells is an urgent need.

Pheophorbide a (Pa), a derivative of chlorophyll a, is an active component from an ethnopharmacological herb *Scutellaria barbata *in China. In previous study, it was shown to exhibit anti-tumor effect on human lung and liver cancers cells [10,11]. Furthermore, we have demonstrated the anti-cancer effects of Pa mediated PDT (Pa-PDT) in human hepatoma and uterine sarcoma cells [12,13]. Meanwhile, its inhibitory effect was also reported in a number of other human cancer cells, such as Jurkat leukemia, pigmented melanoma, colonic cancer and pancreatic carcinoma [14-17].

Although PDT has been suggested in a number of studies as an alternative treatment to overcome MDR [18-23], the approach of Pa-PDT has not yet been evaluated systematically in any human cancer cell model carrying MDR. In this study, we examined the effect of Pa-PDT on cytotoxicity in human hepatoma cells with MDR, namely R-HepG2 cells. Our results demonstrated that Pa-PDT not only induces mitochondrial-mediated apoptosis, but also inhibits MDR by down-regulation of P-glycoprotein activity and expression in R-HepG2 cells.

## Results

### Pa-PDT circumvents drug resistance of R-HepG2 cells

**Figure 1:**
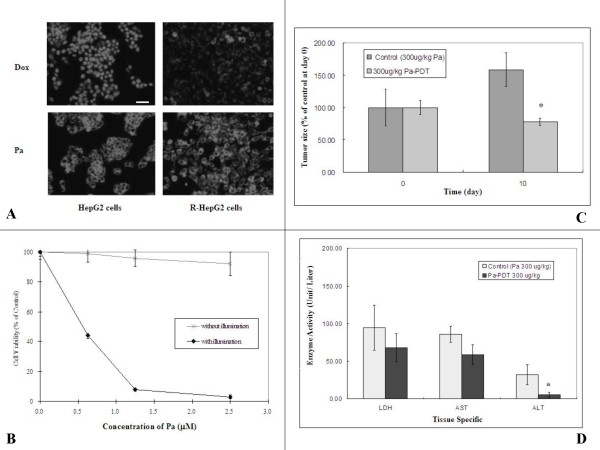
**Pa-PDT circumvents MDR on resistant human hepatoma cells R-HepG2**. **(A) **Intracellular accumulations of Pa and Dox in HepG2 and R-HepG2 cells. Cells (3 × 10^5^/well) were incubated with Pa (2 μM) or Dox (2 μM) for 4 h, and then washed with PBS for 3 times. Fluorescence micrographs were acquired with an excitation wavelength at 400–440 nm and an emission wavelength at 590–650 nm under a Nikon TE2000 fluorescence microscope. Images are representative results of 3 independent experiments. Bar: 50 μm. **(B) ***In vitro *cytotoxicity of Pa-PDT in R-HepG2 cells. Cells (1 × 10^4^/well) were incubated with increasing concentrations of Pa in a 96-well plate for 2 h and exposed to light illumination (84 J/cm^2^) for 20 min and subsequently incubated at 37°C, 5% CO2. For the control group, cells were treated with Pa only without light illumination. Cell survival was then assessed by MTT assay 24 h after treatment. Results are mean ± SD of three independent experiments. **(C) ***In vivo *study of Pa-PDT in R-HepG2 cells bearing mice. R-HepG2 cells (1 × 10^7^) were injected into nude mice subcutaneously at day-7. At day 0, 8 nude mice in groups were treated with Pa (300 μg/kg) and photo-activation (30 min) was applied only once to the treatment group 24 h after the Pa pre-treatment. At day ten, the tumor size of each mouse was measured after the Pa-PDT treatment and the data was expressed as mean ± S.D. (n = 8, * p value < 0.05). **(D) **Tissue damages in Pa-PDT treated mice were assessed by an increase in the leakage of tissue specific enzymes (LDH for heart, and AST and ALT for liver) to the blood. The specific enzyme activities of the blood samples of the tested mice were measured and expressed as the mean ± S.D. (n = 8, * p value < 0.05).

The therapeutic potential of Pa-PDT on MDR was demonstrated on multidrug resistant hepatocellular carcinoma human cell line R-HepG2. The fluorescence of Pa and P-glycoprotein substrate Dox in HepG2 and R-HepG2 cells were semi-quantified under fluorescence microscope. Strong fluorescence from Pa was shown in the cytosol of both Pa-treated HepG2 and R-HepG2 cells, whereas strong Dox fluorescence was observed only in HepG2 but not R-HepG2 cells (Figure 1A). Furthermore, the cell viabilities decreased in a dose-dependent manner of Pa-PDT with IC50 value of 0.6 μM in R-HepG2 cells (Figure 1B) and 0.4 μM in HepG2 cells (data not shown) after 24 h. When light illumination was absent, no toxic effect was observed in the R-HepG2 cells treated with Pa only up to a concentration of 2.5 μM (Figure 1B). Our findings suggest that Pa is a potential agent to kill human hepatoma cells even when they have developed MDR.

### Pa-PDT inhibits the growth of R-HepG2 hepatoma xenograft tumor

The therapeutic potential of Pa-PDT on MDR cancer cells was further confirmed with an animal model. The sizes of R-HepG2 tumor in nude mice were monitored after Pa-PDT treatment. As shown in Figure 1C, Pa-PDT (300 μg/kg, s.c.) significantly (p < 0.05) inhibited the growth of R-HepG2 cells, and the tumor size was reduced from 158.60% (control group) to 29.65% (Pa-PDT treated group) 10 days after light illumination. In addition, no significant increase in the tissue specific enzyme activities of liver (ALT, AST) and heart (LDH) in the blood samples of the Pa-PDT treated mice was observed when compared to those of the control group suggesting that damages of liver and heart were not observed after Pa-PDT treatment (Figure 1D). We also observed that intraveneous injection (i.v.) of Pa followed by PDT also did not cause any side effects in these mice (results not shown).

### Pa-PDT induces apoptosis in R-HepG2 cells

**Figure 2:**
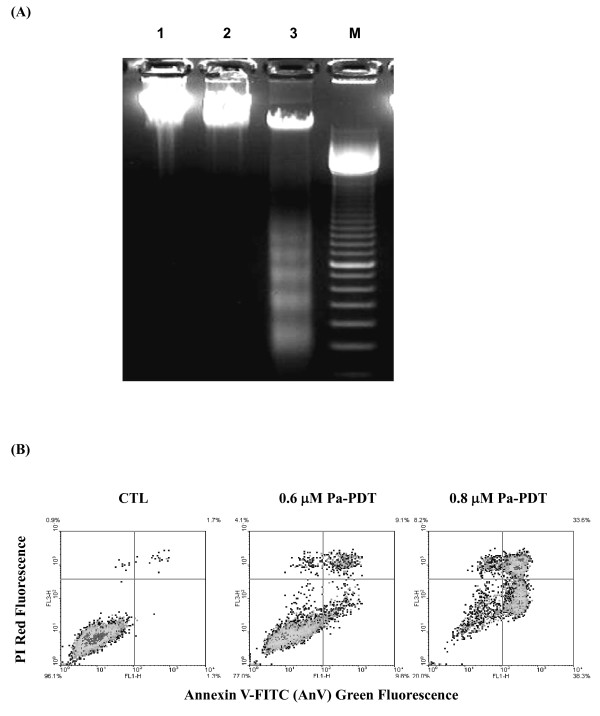
**Pa-PDT induces apoptosis in R-HepG2 cells**. **(A) **DNA fragmentation was revealed by agarose gel electrophoresis. The cells (2 × 10^6^/plate) were collected at 24 h after the treatment, Lane 1: solvent control (0.04% ethanol with light illumination); lane 2: cells treated with Pa (0.6 μM) without light illumination; lane 3: cells treated with Pa (0.6 μM) with 20 min light illumination (84 J/cm^2^); and lane M: DNA markers (100 base pair). **(B) **For the annexin V/PI assay, the Pa-PDT treated cells were collected at 2 h and then stained with annexin V-FITC and PI (15 min) for apoptosis study. Results shown are representative of 3 independent experiments.

The proliferation of tumor cells under chemotherapy can be suppressed by several mechanisms, such as cell cycle arrest, necrosis and apoptosis. In order to determine the underlying mechanism of Pa-PDT, nucleosomal DNA fragmentation was analyzed in R-HepG2 cells with or without the treatment. As shown in Figure 2A, a typical DNA ladder was observed for the 0.6 μM Pa-PDT treated cells. However, no such DNA fragmentation was found in the solvent control with light illumination or samples with Pa but no light illumination (Figure 2A). In order to confirm cell death process, detection of externalization of PS and loss of membrane integrity in R-HepG2 cells were performed. As shown in Figure 2B, 9.8% and 38.3% of early apoptotic cells (annexin V-FITC positive and PI negative) were found in R-HepG2 cells treated with 0.6 μM or 0.8 μM Pa respectively, compared to 1.3% in the control sample. The cell population with late phase of apoptosis (annexin V-FITC positive and PI positive) also increased from 1.7% to 9.1% and 33.6% after the treatment with Pa at 0.6 and 0.8 μM respectively. Our results therefore suggest that Pa-PDT treatment would trigger cell death via apoptosis in the R-HepG2 cells.

### Pa-PDT activates apoptosis via JNK-mediated pathway in R-HepG2 cells

**Figure 3:**
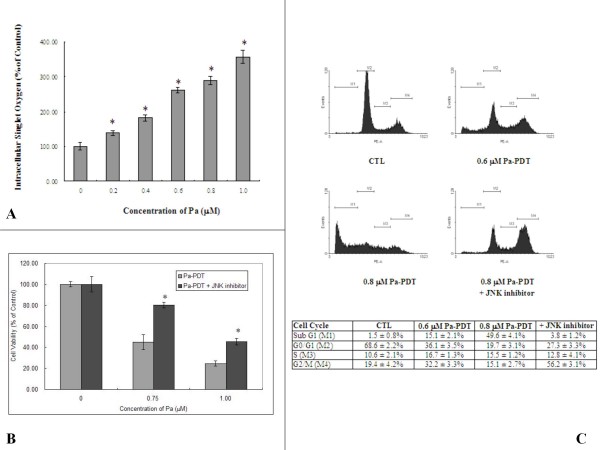
**Pa-PDT triggers JNK-dependent apoptotic pathway in R-HepG2 cells**. **(A) **Intracellular singlet oxygen generation. Cells (2 × 10^4^/well) were treated with various concentrations of Pa-PDT, then the treated cells were stained with 10 μM trans-1-(2'-methoxyvinyl) pyrene at 10 min after the PDT for 15 min in dark. The intensity of fluorescence was detected with a plate reader; the results shown are representative as mean ± S.D. of 3 independent experiments (* p value < 0.005). **(B) **Involvement of JNK in the Pa-PDT induced cell death pathway. Cells (1 × 10^4^/well) were co-incubated with increasing concentrations of Pa and 0.5 μM JNK inhibitor in a 96-well plate for 2 h and then treated with PDT, and subsequently incubated at 37°C, 5% CO_2_. Cell viability was then assessed by MTT assay 24 h after treatment. Results are mean ± SD of three experiments (* p value < 0.005). **(C) **For cell cycle analysis, cells with Pa or co-incubated with 0.6 μM Pa and 0.5 μM JNK inhibitor were harvested at 24 h after PDT treatment, fixed and stained with PI (10 μg/ml, 30 min). The stained cells were analyzed by flow cytometry, and the percentage of cell population in subG1, G0/G1, S and G2/M phase were shown in the table as mean ± SD of three independent experiments.

Singlet oxygen may be released from Pa-PDT-mediated damaged mitochondria [13]. Actually, the intracellular singlet oxygen level was increased in a dose-dependent manner in R-HepG2 cells immediately after Pa-PDT treatment (Figure 3A). As shown in Figure 3B, the cytotoxicity of Pa-PDT was inhibited in the R-HepG2 cells when the cells were pre-treated with JNK inhibitor, where the cell viabilities could be restored to 80.5% and 48.5% in the 0.75 μM and 1.0 μM Pa-PDT treated R-HepG2 cells respectively. The reversal effect of the JNK inhibitor was quantified by PI-staining, the flow cytometric results show that cells from sub-G1 phase accounted for 15.1% and 49.6% of the total cell population with 0.6 μM and 0.8 μM Pa-PDT at 24 h respectively, as compared to 1.5% in the solvent control (Figure 3C). In contrast, no significant increase in sub-G1 population was observed in the Pa-PDT treated cells co-incubated with JNK inhibitor. It showed an induction of G2/M phase arrest instead (Figure 3C). Therefore, a dose-dependent effect was found in R-HepG2 cells after Pa-PDT treatment.

### Pa-PDT inhibits P-glycoprotein-mediated MDR by JNK activation in R-HepG2 cells

**Figure 4:**
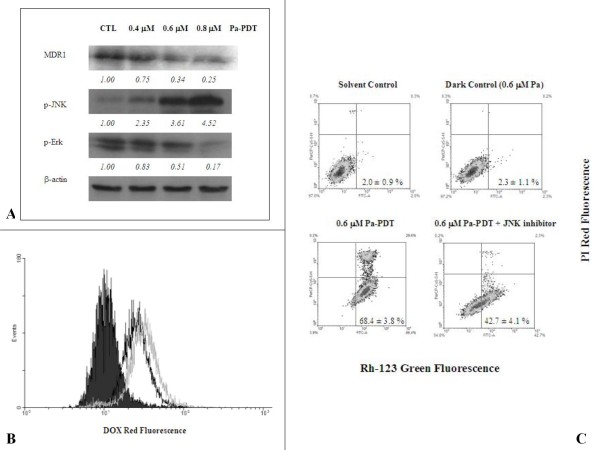
**Pa-PDT inhibits p-glycoprotein mediated MDR in R-HepG2 cells**. **(A) **Differential expression of MDR proteins in Pa-PDT treated R-HepG2 cells. Cells (3 × 10^6^) were treated with Pa alone (0.4 μM Pa without PDT) or (0.4 μM, 0.6 μM, or 0.8 μM) Pa for 2 h and then with light illumination (84 J/cm^2^) for 20 min. Cells were collected at 2 h after PDT treatment, then cell lysates were analyzed using Western blotting. The protein expression levels were semi-quantified and shown as relative intensities normalized with the band intensity of the housekeeping β-actin in each sample. Representative results from a single experiment are shown from 5 independent experiments.**(B) **For the intracellular accumulation of Dox, cells (4 × 10^5^/well) were treated with 0.04% ethanol (CTL, black solid), 0.6 μM (black line) and 0.8 μM (gray line) of Pa-PDT, and then the culture medium was changed to 4 μM Dox and further incubated for 2 h at 37°C, 5% CO2. The cells were collected and the intensity of Dox fluorescence was measured by a flow cytometer. **(C) **For detection of P-glycoprotein activity, R-HepG2 cells (1 × 10^4^/well) were pre-incubated with 0.04% ethanol (solvent control), 0.6 μM Pa (dark control), 0.6 μM Pa-PDT, or 0.6 μM Pa-PDT with 0.5 μM JNK inhibitor in a 6-well plate for 2 h and then the samples were illuminated with PDT. The treated cells were stained with 10 μM Rh-123 for 2 h at 37°C, 5% CO2 and then incubated with 5 μg/ml PI for further 15 min at room temperature. The cells were collected and analyzed by a flow cytometer, where the lower right quadrant (Rh-123 positive and PI negative) represents the cells that have intact plasma membrane but with down-regulated P-glycoprotein activity. The figure is a representative of 5 experiments and the results shown as mean ± SD.

The activation of Mitogen-Activated Protein Kinase (MAPK) enzyme JNK is recently suggested to be involved in the down-regulation of P-glycoprotein [24]. Interestingly, we revealed that the expression of P-glycoprotein was down regulated dose-dependently in R-HepG2 cells at 2 h after Pa-PDT treatment (Figure 4A), with induction of phosphorylated JNK and down-regulation of a PDT-resistant protein p-Erk when compared to the control that was treated with 0.4 μM Pa alone as a dark control of the experiment. This finding was confirmed by the increase in the intracellular level of the P-glycoprotein substrate doxorubicin, which is a commonly used anti-cancer agent in clinic, in R-HepG2 cells treated with 0.6 μM and 0.8 μM Pa-PDT at 2 h (Figure 4B). In addition, co-staining of P-glycoprotein substrate Rh-123 and PI was performed, in order to rule out the possibility that the increase of membrane permeability for the substrate was due to cell death. According to our findings, Pa-PDT treatment could suppress the P-glycoprotein activity in a dose-dependent manner (Figure 4C), as the intensity of Rh-123 in the R-HepG2 cells with intact membrane (PI negative) was increased in a dose-dependent manner from 2.0% (solvent control) and 2.3% (dark control) to 68.4% in the cells treated with 0.6 μM Pa-PDT. In addition, the Pa-PDT mediated inhibition on P-glycoprotein was reversed by JNK inhibitor, as the intensity of Rh-123 was found to be 42.7% in 0.6 μM Pa-PDT treated R-HepG2 cells in the presence of JNK inhibitor (Figure 4C). Thus, Pa treatment is able to inhibit the P-glycoprotein mediated MDR via JNK activation when PDT illumination was applied in R-HepG2 cells.

### Pa-PDT triggers cell-death via mitochondrial apoptotic pathway in R-HepG2 cells

**Figure 5:**
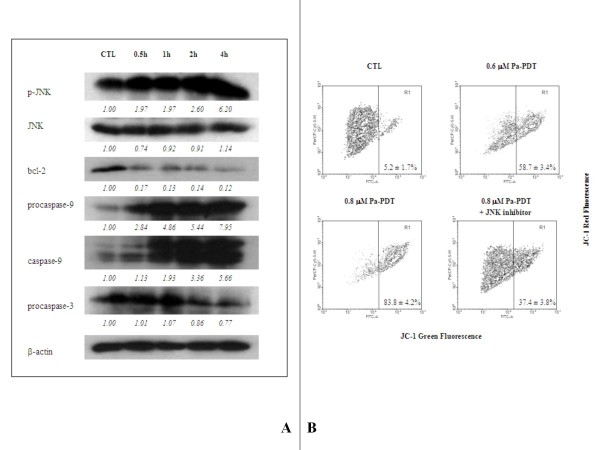
**Pa-PDT activates JNK-mediated apoptosis in R-HepG2 cells**. **(A) **Changes in apoptosis regulatory proteins in Pa-PDT treated R-HepG2 cells. Cells (3 × 10^6^) were treated with solvent (0.04% ethanol (CTL)) or 0.6 μM Pa-PDT, and collected at appropriate time points, where solvent control was collected at 30 min after the treatment. The cell lysates were prepared and the changes in the level of various apoptosis-related proteins were analyzed using Western blotting. The protein expression levels were semi-quantified and shown as relative intensities normalized with the band intensity of the housekeeping β-actin in each sample. Representative results from a single experiment are shown from 5 independent experiments.**(B) **To determinate the effect of Pa-PDT on Δψm, flow cytometry analysis was conducted in R-HepG2 cells (3 × 10^5^/well) 1 h after light illumination (84 J/cm^2^, 20 min) with 0.6 μM Pa, 0.8 μM Pa, or 0.8 μM Pa with 0.5 μM JNK inhibitor. The cells were then stained with JC-1 (10 μM) for 15 min. The green and red fluorescence of JC-1 were acquired subsequently with a flow cytometer, and the results shown as mean ± SD of 3 independent experiments.

Apoptosis can be initiated by JNK that triggers the intrinsic (mitochondrial-dependent) pathways which result in DNA fragmentation. According to results in Figure 5A, phosphorylation of JNK was observed at 30 min after Pa-PDT treatment, where the native form of JNK was slightly decreased. The Pa-PDT induced p-JNK caused down-regulation of the pro-apoptotic protein bcl-2 that facilitates the collapse of mitochondrial membrane and eventually initiates the intrinsic apoptotic pathway. Our results support this notion where both procaspase-9 and the active caspase-9 were significantly increased in the Pa-PDT treated R-HepG2 cells, and finally trigger the activation of procaspase-3 that was started to be cleaved at 2 h after the Pa-PDT treatment (Figure 5A). The involvement of JNK-mediated intrinsic apoptotic pathway was confirmed by the change of mitochondrial membrane potential in Pa-PDT treated R-HepG2 cells. The cell population with depolarized mitochondrial membrane was increased dose-dependently from 5.2% to 83.8% in 0.8 μM Pa-PDT treated cells; however, such collapse was partially inhibited in the presence of JNK inhibitor (Figure 5B). Our finding demonstrated that the Pa-PDT mediated death of R-HepG2 cells is a result of the activation of JNK that initiates the mitochondrial-mediated apoptotic pathway.

## Discussion

MDR is a frequent phenomenon in human liver cancers with prolonged chemotherapy treatment. Hepatoma cells with MDR property are able to tolerate a number of anti-tumor agents [25]. Although using an increasing dosage of drugs may overcome MDR, the side effects of drugs usually shorten the life period of patients. PDT is a potential method to overcome MDR [18-22], which relies on a synergistic effect of two non-toxic elements, photosensitizer and light irradiation [3,4].

The anti-tumor effects of Pa-PDT were reported in skin cancer and hepatoma *in vitro *[23,26]. However, its therapeutic potential in killing human liver cancer especially those with MDR is not fully investigated. A reduction of intracellular drug concentration in tumor cells with MDR is a common phenomenon for the anti-cancer agents that belong to the P-glycoprotein substrate family. This mechanism decreases the drug effect and drug sensitivity in patients during chemotherapy [25]. According to the results shown in Figure 1A, the cellular uptake levels of 2 μM Pa in both HepG2 and R-HepG2 cells were similar, whereas a reduction was observed in the case of 2 μM Dox in R-HepG2 cells as a result of P-glycoprotein overexpression. This result is consistent with our previous finding where higher concentrations of drugs, nearly 20 μM Pa and 8 μM Dox were applied and the drugs uptake levels were monitored by flow cytometry [27]. In addition, the cytotoxicity of Pa-PDT was measured in human hepatoma cells HepG2 and its MDR line R-HepG2. As shown in Figure 1B, Pa-PDT could circumvent the MDR of R-HepG2 cells and killed the cells with an IC50 value of 0.6 μM, only 1.5-fold higher than that of its parental cell line (data not shown). The high degree of cytotoxicity in R-HepG2 cells might be a result of the alternations in the apoptotic mechanism that render the cells more sensitive to Pa-PDT [28]. In addition, our results demonstrate that the cytotoxicity of Pa was rapidly increased when light illumination was applied. For example, the IC50 value of Pa was dramatically decreased from 25 μM (from our previous study) to 0.6 μM at 24 h after the PDT treatment in R-HepG2 cells (Figure 1B) [27]. Pa shares the same characteristics with other photosensitizers in that no cell death was induced when cells are treated with light illumination or Pa (at concentrations below 2 μM) alone, this property would minimize the undesirable side effects towards normal tissues during anti-tumor treatments. To show the therapeutic potential of Pa-PDT in hepatoma cells with MDR, *in vivo *study was performed. Our results illustrate that Pa-PDT could significantly (p < 0.05) inhibit the growth of R-HepG2 cells in the tested group with insignificant damages towards the heart and liver of the hosts when compared to that of the control in which only Pa was injected and no light irradiation was applied (Figure 1C and 1D). The level of ALT was decreased in the Pa-PDT treated group. Pa is an active component of a traditional Chinese medicine *S. barbata *that is commonly used for treating liver disease, our finding thus suggests a potential beneficial effect of Pa-PDT on liver protection and further study should be conducted to confirm this idea [11]. Nevertheless, our previous study showed that Pa-PDT is less cytotoxic in the human normal hepatic cell line WRL-68 *in vitro*, and *in vivo *study reported the short retention of Pa in normal tissues as HDL and LDL can significantly promote the efflux of Pa from normal cells [12,29].

The mechanism of Pa-PDT mediated anti-proliferative effect in R-HepG2 cells was investigated in the present work. Apoptosis is important for tissue homeostasis and cell death. Our previous study demonstrated that Pa induced cell cycle arrest in R-HepG2 cells when no light illumination was applied [27]. Our present results suggest that PDT treatment could render Pa to become an apoptosis inducer for R-HepG2 cells as evidenced by the following observations: DNA fragmentation, a hallmark phenomenon of apoptosis [30], was only detected in Pa-treated samples with light illumination (Figure 2A); and the level of PS externalization (Figure 2B) were dramatically increased in the Pa-PDT treated R-HepG2 cells.

Many proteins are involved in the apoptosis induction and execution processes [4,31,32]. For example, activation of caspase cascade is one of the major effectors for promoting apoptosis. Our observation showed that the fluorescence of Pa was distributed only in cytoplasm but not in nuclei indicating that Pa largely acts on the intrinsic apoptotic pathway (Figure 1A). To support this notion, our results clearly demonstrated that the active form of caspase-9 was increased and the precursor of caspase-3 was cleaved significantly after Pa-PDT treatment (Figure 5A). It implies that the mitochondrial apoptotic pathway might be activated by Pa-PDT treatment in R-HepG2 cells. Putting all our results together, it seems likely that the activated caspase-9 acted on pro-caspase-3 and the activated caspase-3 cleaved PARP to prevent DNA repair and trigger the chromosomal DNA fragmentation in R-HepG2 cells, leading to cell death.

To explain the involvement of mitochondria in the mechanism of Pa-PDT, we hypothesized that Pa works like other photosensitizers which can cause damages of mitochondria in tumor cells after PDT [24]. In fact, the intracellular singlet oxygen concentration increased in R-HepG2 cells after Pa-PDT (Figure 3A) and this increase may be due to mitochondrial damage. Intracellular location of the photosensitizer could affect the efficiency of the killing effect. In our previous study, we have demonstrated that Pa was localized to the mitochondria in the treated cells to elicit the photodamage [12,13]. In this study, our data further confirm this finding, as a collapse of the mitochondria membrane potential was detected by the JC-1 assay (Figure 5B). In addition, the decrease in the level of the anti-apoptotic protein bcl-2 would also elicit more mitochondrial injuries (Figure 5A) which facilitate the release of mitochondrial singlet oxygen to the cytosol and lead to the rapid increase of intracellular singlet oxygen concentration in Pa-PDT treated cells (Figure 3A). Therefore, our observations support the principle that photosensitizers, including Pa, can cause damage of mitochondria of tumor cells after PDT, and this triggers cell death pathways, including the release of singlet oxygen, etc., in the treated tumor cells [4].

A number of proteins respond to the internal induced stresses. One example is the MAPK kinase JNK, which is found to be activated after Pa-PDT treatment (Figure 5A). Application of JNK inhibitor on Pa-PDT treated R-HepG2 cells would suppress the induction of apoptosis and shift the cells to undergo cell-cycle arrest at G2/M phase (Figure 3C). The Pa-PDT induced apoptosis is started by the collapse of mitochondria which is facilitated by JNK, according to Figure 4B, the degree of mitochondrial membrane potential change was reduced significantly when activation of JNK was inhibited. Our results showed that Pa-PDT suppresses the proliferation of R-HepG2 cells by activating the JNK-mediated intrinsic apoptotic pathway.

In addition, the activated JNK (p-JNK) would inhibit the expression of P-glycoprotein and result in the suppression of MDR property on the cancer cells [24,33]. PDT is recently reported to reduce the expression of P-glycoprotein in cancer cells, however, the linkage of JNK pathway and PDT-induced down-regulation of P-glycoprotein is not yet investigated [34]. The expression of P-glycoprotein was significantly decreased after Pa-PDT treatment (Figure 4A) and led to a decreased refluxing of the P-glycoprotein substrates Dox and Rh-123 in R-HepG2 cells (Figure 4B and 4C). Such inhibition can be suppressed by co-incubating with JNK inhibitor (Figure 4C). Although our previous study reported that 20 μM Pa alone (without light illumination) can also inhibit the P-glycoprotein expression in R-HepG2 cells, the response time is much longer (24h-incubation with Pa alone) and the mechanism is due to the regulation of MDR1 expression on transcriptional level instead of the activation of JNK [28]. Furthermore, we showed that the activation of Erk, responsible for the resistance to PDT, was suppressed in the Pa-PDT treated R-HepG2 cells (Figure 4A); it implied that using Pa as a photosensitizer will not induce resistance to the photodynamic treatment [35]. Taken together, our findings provide the first evidence that Pa-PDT would decrease the P-glycoprotein level via JNK-mediated pathway, and result in an enhancement of intracellular Dox level in MDR human liver tumor cells which would overcome the difficulty in clinical situation.

## Materials and methods

### Materials

Culture media were purchased from Invitrogen (USA). All other chemicals were purchased from Sigma Chemical Co (USA). Pa originally purified from *Scutellaria barbata *[11] is now commercially available. Pa used in this study was purchased from Frontier Scientific (USA), and JNK inhibitor (JNK inhibitor II) was purchased from Merck (USA).

### Cell cultures

HepG2 cells obtained from American Type Culture Collection (ATCC) were cultured in RPMI-1640 medium with 10% (v/v) fetal bovine serum (FBS), 100 U/ml penicillin G and 100 μg/ml streptomycin. Cells were incubated at 37°C in a humidified atmosphere of air/CO2 (95%: 5%). For the development of MDR cells, HepG2 cells were cultured with Dox from 0.1 to 100 μM during cell passages. After several rounds of selection, R-HepG2 with MDR properties was obtained. R-HepG2 was developed in our laboratory and characterized with an overexpression of P-glycoprotein [6,24]. R-HepG2 cells were then cultured with 1.2 μM Dox to maintain its MDR properties [25].

### Measurement of drug accumulation

The method described earlier was used [25]. Cells (3 × 10^5^/well) grown on the coverslips were preloaded with 2 μM Pa or 2 μM Dox and incubated at 37°C in a humidified atmosphere of air/CO2 (95%: 5%) for 2 h without PDT treatment. After washing 3 times with PBS, fluorescence of Pa or Dox was observed under a Nikon TE2000 fluorescence microscope with an excitation wavelength at 578 nm and an emission wavelength at 610 nm.

### Pa-mediated photodynamic therapy (Pa-PDT) on cancer cells

Cells were seeded in culture plate and incubated for 24 h to allow attachment. After washing with phosphate buffered saline (PBS), the cells were pre-incubated with appropriate concentrations of Pa for 2 h. Then the cells were photo-irradiated for 20 min using a 600 W quartz-halogen lamp with a long pass filter at 610 nm, and the light intensity was 70 mW/cm^2^(i.e. 20 min of irradiation = 84 J/cm^2^) [13].

### Measurement of cell viability

Twenty-four hours after Pa-PDT treatment, the cells were washed gently with PBS. Thirty microliters of methyl-thiazoldiphenyl tetrazolium (MTT) (5 mg/ml) was added to each well and incubated for 2 h at 37°C. The MTT solution was then replaced by 100 μl of dimethyl sulfoxide in each well. Absorbance was measured with a microtitre-plate reader (Bio-Rad) at 540 nm and all data were calculated as percentage of control.

### In vivo study of Pa-PDT on R-HepG2 cells bearing nude mice

The method described earlier was used with slight modifications [12]. Male 4–6 weeks old nude mice were supplied by the Laboratory Animal Services Center of The Chinese University of Hong Kong. The care and use of the animals were in compliance with institutional guidelines. R-HepG2 cells (1 × 10^7^) were inoculated at the back of nude mice. After one week of tumor growth (tumor size about 150 mm^3^), 16 mice were randomly divided into treatment and control groups. Both groups received 300 μg/kg Pa by local injection (s.c.) nearby the transplanted tumor site only once. After 24 h, one photo-activation (126 J/cm^2^) was applied to whole animal for 30 min in the treatment group. After 10 days, the tumor volume of each subject was determined by caliper measurements (mm) according to the following formula: Volume (mm^3^) = (Length × Width × Height) π/2. The data were calculated as percentage of control.

### Assessment on damages of heart and liver

Blood samples of each tumor bearing nude mouse were collected in heparin-coated tubes at the end of the treatment. After centrifugation at 1500 × g for 5 min at 4°C, the plasma was collected and stored at 4°C for enzyme assays. Lactate dehydrogenase (LDH), aspartate aminotransaminase (AST) and alanine aminotransaminase (ALT) activities were determined by using Stanbio LDH Liqui-UV^® ^kit (Stanbio Laboratory), Infinity™ AST reagent and Infinity™ ALT reagent (Thermo Electron Corporation) respectively. All enzyme assays were carried out at 37°C in a UV1601 spectrophotometer with a TCC-240A temperature controller (Shimadzu Ltd.).

### DNA fragmentation detection

The Pa- or solvent-treated cells (2 × 10^6^/dish) were washed twice with PBS 24 h after PDT as in our previous study [12]. The cells were then resuspended in 400 μl of DNA lysis buffer (200 mM Tris-HCl, 100 mM EDTA, 1% SDS, pH 8.3). The mixture was vortexed vigorously and 20 μl of proteinase K (10 mg/ml) was added. After 2 h of incubation at 37°C, 150 μl of saturated sodium chloride solution was added and mixed completely. The samples were centrifuged at 6500 × g for 15 min at 4°C. Supernatant was collected, mixed with 1 ml of cold ethanol and then centrifuged again at 15000 × g for 20 min. The pellets were washed with 1 ml of cold 75% ethanol, centrifuged at 15000 × g for 20 min and allowed to dry. Twenty microliters of ribonuclease A (RNase A) (0.2 mg/ml) was added to the dry pellet and incubation was allowed at 37°C for 90 min. The pattern of genomic DNA extract was visualized by electrophoresis in 1.2% agarose gel containing ethidium bromide and photographed under UV light.

### Detection of phosphatidylserine (PS) translocation

To detect the translocation of PS, an annexin V-FITC kit (Trevigen) was used. The cells (4 × 10^5^/well) were stained with 100 μl of staining reagent at room temperature for 15 min in dark according to our previous study [13]. Then the samples were mixed with 400 μl of 1× binding buffer and analyzed by FACSort flow cytometry (Becton Dickinson) with the CellQuest software. The cell population was gated by FSC and SSC. The fluorescence signals were acquired by FL1 and FL3 channel in log scale, and no influence of Pa fluorescence was detected when its concentration is lower than 2 μM.

### Detection of intracellular singlet oxygen after Pa-PDT

The assay was performed in a 96-well culture plate containing 2 × 10^4 ^cells in each well. The cells were gently rinsed with PBS at 10 min after PDT, and 30 μl of 10 μM trans-1-(2'-methoxyvinyl) pyrene was added to each well and incubated in dark for 15 min at 37°C. Then, the staining solution in the well was replaced by 50 μl PBS, and the intensities of fluorescence were acquired by a FLUOstar Galaxy plate reader (BMG Labtech).

### Cell cycle analysis

This assay was performed according to the procedures as described in [27]. Cells (3 × 10^5^/well) were seeded in a 6-well plate and incubated for 24 h to allow attachment. Twenty-four hours after photodynamic treatment, the cells were washed twice with PBS and fixed in 70% ethanol overnight at 4°C. Then the cells were resupended in PBS containing propidium iodide (PI) (10 μg/ml) and RNase A (50 μg/ml). The samples were then analyzed by a FACSort flow cytometer (Becton Dickinson).

### Western blot analysis

The method described earlier was used [36]. The PDT-treated cells (3 × 10^6 ^cells/plate) collected at appropriate time after PDT were lysed by incubating the cells in the whole cell extraction buffer (2% sodium dodecyl sufate (SDS), 10% glycerol, 625 mM Tris-HCl (pH 6.8), β-mercaptoethanol (5% v/v)) for 2 h at 4°C. Samples were boiled in water for 10 min, and then centrifuged at 11000 × g for 20 min at 4°C. The supernatant proteins were resolved by 12.5% SDS polyacrylamide gel and transferred to 0.45 μm polyvinylidene fluoride (PVDF) membrane (Immobilon, Millipore). The membrane was blocked with 10% non-fat milk in Tris buffered saline containing Tween-20 (TBS-T) (20 mM Tris-HCl (pH 7.6), 150 mM NaCl, 0.1% Tween-20) and then incubated with primary human antibodies against β-actin (Sigma), Bcl-2, JNK, p-JNK, p-ERK, procaspase-3 (Santa Cruz), caspase-9 (Stressgen), and P-glycoprotein (Merck) in TBS-T. After incubation with the secondary antibody conjugated with horseradish peroxidase, immunodetected proteins were visualized by using an enhanced chemiluminescence assay kit (Amersham Life Science). The protein content of each sample was normalized by the corresponding β-actin.

### Detection of P-glycoprotein activity

The Pa-PDT treated cells (3 × 10^5^/well) on a 6-well plate were incubated with 10 μM Rh-123 following photodynamic treatment. After 2h-incubation at 37°C, the cells were collected and washed twice with PBS, and then resupended in PBS containing PI (5 μg/ml) for 15 min at room temperature. The samples were finally analyzed by the FACSort flow cytometer (Becton Dickinson).

### Detection of changes in mitochondrial membrane potential (Δψm)

Cells (3 × 10^5^/well) were seeded to a 6-well culture plate and photodynamically treated on the next day. The cells were collected and washed twice with PBS 1 h after PDT, and then stained with 10 μg/ml JC-1 with a supplement of 20 μM verapamil for 15 min at 37°C. Then the cells were analyzed by FACSort flow cytometry with the CellQuest software (Becton Dickinson). The cell population was gated by FSC and SSC. The signal was detected by FL1 and FL3 channels for JC-1 in log scale, where no influence of Pa fluorescence was detected when its concentration is lower than 2 μM.

### Statistical analysis

Data were presented as mean ± S.D. of various experiments. Statistical analysis was performed by one-way analysis of variance (ANOVA) with Bonferroni post-hoc test (two-tailed). *P *< 0.05 was considered statistically significant.

## Competing interests

The authors declare that they have no competing interests.

## Authors' contributions

PMKT participated in the design of the study, carried out the work and draft the manuscript. DMZ participated in the design of the apoptosis study. N-HBX participated in Western blotting analyses. SKWT, MMYW, SKK, and WPF read and approved the final manuscript. KPF secured fundings, conceived the study, and participated in its design and coordination and finalized the draft of the manuscript. All authors read and approved the final manuscript.
